# Exploring low grade inflammation by soluble urokinase plasminogen activator receptor levels in schizophrenia: a sex-dependent association with depressive symptoms

**DOI:** 10.1186/s12888-021-03522-6

**Published:** 2021-10-26

**Authors:** Therese Torgersen Bigseth, John Abel Engh, Jens Egeland, Eivind Andersen, Ole Andreas Andreassen, Gry Bang-Kittilsen, Ragnhild Sørum Falk, Tom Langerud Holmen, Morten Lindberg, Jon Mordal, Jimmi Nielsen, Nils Eiel Steen, Thor Ueland, Torkel Vang, Mats Fredriksen

**Affiliations:** 1 Division of Mental Health and Addiction, Vestfold Hospital Trust, Sykehuset i Vestfold, PO Box 2168, 3103 Tonsberg, Norway; 2 Department of Psychology, University of Oslo, PO Box 1094, Blindern 0317 Oslo, Norway; 3 Faculty of Humanities, Sports and Educational Science, University of South-Eastern Norway, PO Box 235, 3603 Kongsberg, Norway; 4 NORMENT, Division of Mental Health and Addiction, Oslo University Hospital & Institute of Clinical Medicine, University of Oslo, Psychosis Research Unit/TOP, Ullevaal Hospital, building 49, PO Box 4956, Nydalen 0424 Oslo, Norway; 5 Oslo Centre for Biostatistics and Epidemiology, Oslo University Hospital, PO Box 4950, Nydalen 0424 Oslo, Norway; 6 Department of Laboratory Medicine, Vestfold Hospital Trust, PO Box 2168, 3103 Tonsberg, Norway; 7 Mental Health Centre Glostrup, Copenhagen University Hospital, Copenhagen, Denmark; 8 Research Institute of Internal Medicine, Oslo University Hospital Rikshospitalet, PO Box 4950, Nydalen 0424 Oslo, Norway; 9 Institute of Clinical Medicine, University of Oslo Faculty of Health Sciences, PO Box 1171, Blindern 0318 Oslo, Norway; 10 K.G. Jebsen TREC, University of Tromso, 9037 Tromso, Norway

**Keywords:** Schizophrenia, Depression, suPAR, Urokinase, CRP, Inflammation, Immunesystem, Biomarker, Sex-difference

## Abstract

**Background:**

There is evidence of increased low grade inflammation (LGI) in schizophrenia patients. However, the inter-individual variation is large and the association with demographic, somatic and psychiatric factors remains unclear. Our aim was to explore whether levels of the novel LGI marker soluble urokinase plasminogen activator receptor (suPAR) were associated with clinical factors in schizophrenia and if such associations were sex-dependent.

**Method:**

In this observational study a total of 187 participants with schizophrenia (108 males, 79 females) underwent physical examination and assessment with clinical interviews (Positive and Negative Syndrome Scale (PANSS), Calgary Depression Scale for Schizophrenia (CDSS), Alcohol Use Disorder Identification Test (AUDIT), and Drug Use Disorder Identification Test (DUDIT)). Blood levels of suPAR, glucose, lipids, and high sensitivity C-reactive protein (hsCRP) were determined and body mass index (BMI) calculated. Multivariable linear regression analyses were used adjusting for confounders, and sex interaction tested in significant variables.

**Results:**

Adjusting for sex, age, current tobacco smoking and BMI, we found that levels of hsCRP and depressive symptoms (CDSS) were positively associated with levels of suPAR (*p* < 0.001). The association between suPAR and CDSS score was significant in females (*p* < 0.001) but not in males. Immune activation measured by hsCRP was not associated with depressive symptoms after adjusting for BMI.

**Conclusion:**

Our findings indicate that increased suPAR levels are associated with depressive symptoms in females with schizophrenia, suggesting aberrant immune activation in this subgroup. Our results warrant further studies, including longitudinal follow-up of suPAR levels in schizophrenia and experimental studies of mechanisms.

**Supplementary Information:**

The online version contains supplementary material available at 10.1186/s12888-021-03522-6.

## Background

Involvement of the immune system in the pathogenesis of schizophrenia has been investigated for several decades [1]. In this regard, immune-mediated mechanisms seem to be relevant in the prenatal stage and also through childhood and in adolescence and adulthood [2, 3]. Furthermore, immune disorders such as non-neurological autoimmune diseases are associated with increased risk of psychosis [4]. Genetic variants in the immune system have also been implicated in the etiology of schizophrenia, at least in a subgroup of patients (PGC Nature 2014).

The overlap of symptoms in current diagnostic classification makes research, diagnostics and treatment challenging [5]. Previous studies have suggested the existence of subgroups with immune-dysregulation in schizophrenia [1, 6], and other severe mental disorders such as bipolar disorder [7] and major depressive disorder (MDD) [8, 9]. Further, analyses of postmortem endothelial cells from schizophrenia patients with increased inflammatory burden have revealed transcriptional alterations associated with endothelial cell dysregulation [10].

As a biomarker of inflammation, C-reactive protein (CRP) has received much attention. Numerous studies have evaluated levels of CRP as a means of identifying inflammatory subgroups in schizophrenia, mostly reporting modestly but significantly elevated levels mainly related to the severity of symptoms occurring during the relapsing phase [11]. However, despite being a robust biomarker, its role in schizophrenia is not yet established. The association between CRP and central obesity and infections further complicates interpretation of CRP in the context of schizophrenia. Thus, identification of biomarkers linked more specifically to psychological state and pathophysiological processes is warranted.

The urokinase plasminogen activator receptor (uPAR) is a glycoprotein active across several systems (e.g. the fibrinolytic and inflammatory systems). Linked to the cell surface via a glycosyl phosphatidylinositol anchor, uPAR is found on a variety of cells, e.g. immune cells, endothelial cells and neurons and involved in numerous inflammatory processes with effects on development of axons, brain development and maturation as well as neuro repair and neuroprotection [12–14]. Upon immune activation, uPAR can be shed from the plasma membrane, and the resulting soluble uPAR (suPAR) can easily be measured in blood samples. The suPAR protein displays robust pre-analytic characteristics with regard to sampling, storage and freeze-thaw cycles [15] as well as stability beyond fasting and circadian rhythm [16]. LGI involving suPAR is mainly associated with endothelial dysfunction [12]. However, a small study found the suPAR gene (PLAUR) to be upregulated in visceral fat in non-obese patients with depression and/or anxiety [17].

Large population-based studies have revealed a positive association between blood levels of suPAR and the following factors; female sex, increasing age, unhealthy lifestyle, cardiovascular risk factors, diabetes, as well as low socioeconomic status [18–20]. Additionally suPAR levels are elevated in patients with MDD [21–24].

Levels of suPAR were significantly increased in heterogeneous samples of schizophrenia patients e.g. including both sexes, alcohol and drug users as well as somatic diseases [25, 26]. In contrast, no difference was found between a homogenous males sample with acute phase schizophrenia and healthy controls [27]. However, neither of these studies thoroughly investigated the potential associations between suPAR levels and clinical characteristics, which may identify clinical subgroups as suggested for other immune mechanisms [6, 28].

Since differentiation of both the immune system and the central nervous system (CNS) reveal sex differences, cross-talk between these two systems could contribute to the sex differences observed in symptoms, cognition and clinical features (e.g. age of onset, trajectory) in patients with schizophrenia [29, 30]. Sex differences in suPAR levels have consistently been reported in larger population studies [18, 20]. Due to the large heterogeneity in schizophrenia, identification of subsets of patients could lead to higher precision in experimental studies of underlying mechanisms as well as more individualized diagnostics and treatment. The suPAR protein seems to have potential to contribute to such subset identification.

In the present study we aimed to identify whether clinical factors, such as psychiatric symptoms and cardiovascular risk factors, were associated with low grade inflammation (LGI) measured by suPAR levels in participants with schizophrenia. Secondly, we wanted to investigate whether associations between clinical factors and suPAR were sex-dependent.

## Methods

### Participants

Participants were recruited in the period 2003–2017 into the collaborating projects Effects of Physical Activity in Psychosis study (EPHAPS) [31] and Thematically Organized Psychosis (TOP) Research project/NORMENT (Norwegian Centre for Mental Disorder Research) [7]. The recruitment was mainly from outpatient psychiatric clinics from the southeast region of Norway. The study was observational and the main inclusion criterion was fulfilling the Diagnostic and Statistical Manual of Mental Disorders (DSM) criteria for schizophrenia spectrum disorder (4th and 5th edition), confirmed by the Structured Clinical Interview for DSM-IV (SCID-I). The participants were 18–67 years of age, understood and spoke a Scandinavian language, and had no mental retardation. In order to exclude participants with severe ongoing and acute infections, we excluded participants with serum levels of CRP above 20 mg/L. Most of the current participants were included as cases in a previous case-control study of suPAR levels (Bigseth et al. 2021). However, in the current study we did not exclude participants with comorbid chronic infectious and autoimmune diseases to reflect a more naturalistic sample.

### Assessments

Information and assessments were obtained or carried out by trained clinicians. Diagnosis was confirmed using the Structured Clinical Interview for DSM-IV axis I Disorders, SCID-I, [32] and the trained clinicians in both research groups (TOP/NORMENT and EPHAPS) underwent a SCID-I training program lead by experts from the University of California Los Angeles (UCLA). Information on sociodemographics, medication, mental and physical health was obtained through patient charts, self-reports and interviews. For baseline assessment of psychotic symptom levels, we used positive and negative subscale of the Positive and Negative Syndrome Scale (PANSS) [33]. The Calgary Depression Scale for Schizophrenia (CDSS) [34], as well as the depression dimension (PANSS depressed factor) in the five factor model of PANSS [35] were used to assess severity of depressive symptoms. A cutoff score CDSS ≥6 was used for depression [34, 36]. Antipsychotic medication doses were quantified by defined daily doses (DDD) according to WHO standards (http://www.whocc.no/) and categorized in either “no medication, low, moderate or high metabolic risk” (see Supplementary Table C, Additional File 3) [37]. We applied the AUDIT to assess alcohol use, and participants were categorized into a group of “problematic use of alcohol” when scores were above defined cutoff values (≥ 5 for females and ≥ 8 for males). DUDIT was applied to assess substance use, where “problematic use of drugs” was defined by cutoff values (≥ 2 for females and ≥ 6 for males) [38]. We used standardized assessment of blood pressure, and body mass index (BMI) was calculated based on standardized measurement of weight and height.

### Blood samples

Fasting blood samples were collected in the morning and subsequently analyzed according to pre-defined protocols. Soluble uPAR and hsCRP were measured in duplicate using a commercially available enzyme-immunoassay (RnDSystems, Stillwater, MN, USA) in a 384-well format using the combination of a SELMA (Jena, Germany) pipetting robot and a BioTek (Winooski, VT, USA) dispenser/washer. Absorption was read at 450 nm with wavelength correction set to 540 nm using an ELISA plate reader (Bio-Rad, Hercules, CA, USA). Intra- and inter-assay coefficients of variation were < 10% [25].

Blood triglycerides, HDL and glucose were analyzed according to standardized procedures in the hospital lab where blood was sampled. We used the Atherogenic Index of Plasma ((AIP) = log(triglycerides/HDL-cholesterol)) as a proxy for cardiovascular disease (CVD) risk [39–41] and fasting glucose as a proxy for diabetes risk (Table 2).

### Statistics

Descriptive statistics of demographic and clinical variables were presented as frequencies and proportions for categorical data and mean and standard deviation (SD) or median and interquartile range (IQR) for continuous data.

To identify factors associated with suPAR levels, we performed linear regression analyses. Variables with established association with suPAR (i.e. sex, age, current tobacco smoking and BMI) were included in the model regardless of the association with suPAR in our sample [18, 42] and hsCRP was used to adjust for inflammatory activity linked to different inflammatory pathways [12]. Because of the known association between age and LGI and a wide age range of included participants, age was adjusted for as a continuous variable, as was BMI and hsCRP. Due to the restricted sample size variables with less evidence from the literature (i.e. PANSS positive, PANSS negative, CDSS, hsCRP, AIP, fasting glucose, blood pressure, problematic use of alcohol and drugs, level of education and antipsychotic medication (DDD and metabolic risk level)) were included into the model according to the purposeful selection approach [43]. In brief, variables were included in the multivariable model if univariable analyses showed *p* < 0.1. Then the variables were removed one at a time, the one with the largest *p*-value first, until all remaining variables were statistically associated with the suPAR level. No outliers were identified. All continuous variables were examined and linearity found satisfactory. We observed no multicollinearity between the independent variables. Results are presented as beta coefficients with 95% confidence intervals (CI) and *p*-values.

To explore the possible effect modification by sex we tested for interactions, on the multiplicative scale, between sex and all the included variables in the final model. In the presence of a significant interaction, we conducted stratified analysis by sex.

Several sensitivity analyses were performed to assess the robustness of the results. To explore the impact of the measurement tool, we substituted the CDSS sum score by the CDSS cutoff score of ≥6 and subsequently PANSS depressed factor. Studying the dimensions of CDSS, we replaced CDSS sum score with each separate item of CDSS in the final model stratifying by sex. To compare effects of inflammation associated with endothelial dysfunction (suPAR) and inflammation associated with central obesity and acute infection response (hsCRP), we explored the relationship between hsCRP and depressive symptoms for the whole sample and stratified by sex. Post-hoc we investigated how levels of suPAR could predict depression in schizophrenia in males and females by constructing a Receiver Operating Characteristic (ROC) curve, defining CDSS ≥6 as positive cases. Psychometric properties of the CDSS, such as Cronbach’s alpha of internal consistency as well as associations between measured symptoms are presented in Supplementary Text 1, Additional File 4 and Supplementary Table B1 and B2, Additional File 2.

Associations with *p* < 0.05 (two-tailed) were considered significant in the main analyses, while the significance level was set to 0.01 in additional analyses to reduce the likelihood of type-I error. All statistical analyses were performed in SPSS version 25 and STATA SE15.

## Results

### Participants characteristics and sex differences

The naturalistic schizophrenia sample consisted of both males (*n* = 108) and females (*n* = 79), mean 32.6 years of age (range 18–67), and included participants with comorbid alcohol and drug use. Females scored higher than males on depression symptom scales, both the CDSS sum score and the CDSS cutoff value (CDSS ≥6) (Table 1). There were higher PANSS general scores in females compared to males (mean difference = 2.70), in particular for PANSS depressed factor (mean difference = 1.84).

**Table 1:** Sociodemographic and psychiatric characteristics of participants with schizophrenia

Characteristics	Total sample (*n* = 187)	Males (*n* = 108)	Females (*n* = 79)
*Sociodemographic features*			
Age [years], mean (SD)	32.6 (12.4)	32.0 (11.6)	33.5 (13.4)
^a^Level of education			
Low, n(%)	90 (48.1)	51 (47.2)	39 (49.4)
Medium, n(%)	73 (39.0)	43 (39.8)	30 (38.0)
High, n(%)	24 (12.8)	14 (13.0)	10 (12.7)
Ethnicity (caucasian), n(%)	177 (94.7)	102 (94.4))	75 (95.0)
Current tobacco smoking, n(%)	102 (54.8)	60 (56.1)	42 (53.2)
^b^Problematic use of alcohol, n(%)	54 (30.0)	29 (27.9)	25 (32.9)
^b^Problematic use of drugs, n(%)	25 (13.9)	15 (14.4)	10 (13.2)
*Psychiatric characteristics*			
PANSS positive, mean (SD)	15.8 (5.2)	15.4 (4.8)	16.3 (5.6)
PANSS negative, mean (SD)	17.5 (6.4)	18.1 (5.8)	16.6 (7.1)
PANSS general, mean (SD)	34.2 (8.7)	33.0 (7.8)	35.7 (9.7)
PANSS total, mean (SD)	67.5 (16.5)	66.6 (15.1)	68.7 (18.3)
PANSS depressed factor, mean (SD)	8.2 (3.3)	7.5 (3.1)	9.3 (3.4)
CDSS sum score, mean (SD)	5.4 (5.2)	4.3 (3.9)	7.0 (6.2)
CDSS ≥6, n(%)	73 (42.0)	34 (34.0)	39 (52.7)
^c^Duration of illness [years], median (IQR)	6.0 (2.0–14.0)	5.0 (2.0–13.8)	7.0 (2.0–14.5)
Admitted to hospital, n(%)	59 (32.4)	35 (33.3)	24 (31.2)

*Note: SD* standard deviation, *IQR* interquartile range (first quartile-third quartile). *CDSS* Calgary Depression Scale for Schizophrenia (0–27), *PANSS* Positive And Negative Syndrome Scale (30–210), Missing data (above 5% of data points): CDSS *n* = 13, Duration of illness: *n* = 18

^a^Categorized as low (less than completed high school), medium (high school completed) and high (3 years or more of college or university education)

^b^Problematic use of alcohol when above defined cut-off values AUDIT (≥5 for females and ≥ 8 for males) and Problematic use of drugs when above defined cut-off values for DUDIT (≥2 for females and ≥ 6 for males)^c^Duration of illness was calculated as age at inclusion minus age at onset of first psychotic episode

^1^t-test, ^2^Mann-Whitney U-test¸^3^Chi-squared test

Clinical somatic characteristics, blood indices and antipsychotic medication are presented in Table 2. Females had lower AIP (mean difference = 0.15) and systolic (mean difference = 9.42) and diastolic blood pressure (mean difference = 4.23) compared to males. Levels of suPAR were higher in females compared to males (mean difference = 0.29).

**Table 2:** Somatic characteristics in our sample of participants with schizophrenia

Characteristics	Total sample (*n* = 187)	Males (*n* = 108)	Females (*n* = 79)
*Somatic features*			
Body Mass Index[kg/m^2^], mean (SD)	28.5 (6.1)	28.1 (5.8)	29.0 (6.5)
Systolic blood pressure [mmHg], mean (SD)	126.1 (15.7)	130.2 (16.3)	120.8 (13.3)
Diastolic blood pressure [mmHg], mean (SD)	79.8 (10.6)	81.7 (11.0)	77.4 (9.6)
*Blood indices*			
suPAR [ng/ml], mean (SD)	1.8 (0.6)	1.7 (0.5)	2.0 (0.6)
hsCRP [mg/L], mean (SD)	2.2 (1,5)	2.2 (1.4)	2,3 (1.5)
HDL cholesterol [mmol/L], mean (SD)	1.2 (0.4)	1.1 (0.3)	1.4 (0.4)
LDL cholesterol [mmol/L], mean, (SD)	3.0 (1.1)	3.0 (1.1)	3.0 (1.1)
Triglycerides [mmol/L], median (IQR)	1.4 (0.9–2.2)	1.5 (1.0–2.6)	1.3 (0.9–1.9)
Fasting glucose[mmol/L], mean (SD)	5.3 (0.9)	5.3 (0.8)	5.2 (1.1)
Atherogenic index of plasma, mean (SD)	0.1 (0.3)	0.2 (0.3)	0.0 (0.3)
*Comorbid diseases*			
Cardiovascular disease, n (%)	21 (11.9)	13 (13.0)	8 (10.4)
Diabetes type II, n (%)	7 (4.0)	3 (3.0)	4 (5.2)
Infectious and autoimmune diseases, n (%)	12 (6.8)	7 (7.0)	5 (6.5)
*Antipsychotic medication*			
Antipsychotic medication [DDD], mean (SD)	1.3 (1.0)	1.3 (1.0)	1.3 (1.0)
^a^Antipsychotic metabolic risk			
No antipsychotic medication, n (%)	20 (10.7)	12 (11.1)	8 (10.1)
Low level, n (%)	27 (14.4)	15 (13.9)	12 (15.2)
Moderate level, n (%)	76 (40.6)	39 (36.1)	37 (46.8)
High level, n (%)	64 (34.2)	42 (38.9)	22 (27.8)

*Note. suPAR* soluble urokinase plasminogen activator receptor, *hsCRP* high sensitivity C-reactive protein, *SD* standard deviation, *IQR* interquartile range (first quartile-third quartile), *DDD* defined daily doses

Missing data (above 5% of data points): BMI: *n* = 12, Systolic and diastolic blood pressure: *n* = 9, HDL-cholesterol: *n* = 16, LDL-cholesterol: *n* = 22, Triglycerides: *n* = 15, Fasting Glucose: *n* = 17, Cardiovascular disease: *n* = 10, Diabetes type II: *n* = 10, Infectious and autoimmune disease: *n* = 10

^a^Antipsychotic metabolic risk: See Table C in supplementary material

### Associations between suPAR and clinical factors

In the multivariable analyses we found that suPAR levels were positively associated with female sex, age, current tobacco smoking, hsCRP and depressive symptoms (CDSS sum score). In addition, BMI was negatively associated with suPAR in the multivariable analyses (Table 3). We found a statistically significant interaction between sex and CDSS sum score (*p* = 0.03) and re-ran the final model, stratified by sex. In males we found positive association between suPAR levels and age, current tobacco smoking and hsCRP, while BMI was negatively associated with suPAR. Depressive symptoms however, were not associated with suPAR in males. For females on the other hand, depressive symptoms and current tobacco smoking were positively associated with suPAR, but age, hsCRP and BMI were not.

**Table 3:** Factors associated with suPAR (ng/ml) in participants with schizophrenia

	Univariable regression			Multivariable regression (*n* = 163)^a^			Multivariable regression Males (*n* = 91)^a^			Multivariable regression Females (*n* = 72)^a^		
	β	95% CI	*p*	β	95% CI	*p*	β	95% CI	*p*	β	95% CI	*p*
Female sex	0.29	0.13 to 0.46	0.001	0.26	0.11 to 0.40	0.001	–	–	–	–	–	–
Age (per 10 years)	0.08	0.01 to 0.15	0.02	0.09	0.03 to 0.15	0.003	0.11	0.03 to 0.19	0.006	0.08	−0.02 to 0.18	0.11
Tobacco smoking	0.33	0.17 to 0.49	< 0.001	0.30	0.16 to 0.45	< 0.001	0.20	0.01 to 0.38	0.04	0.39	0.16 to 0.63	0.001
hsCRP (mg/L)	0.13	0.08 to 0.18	< 0.001	0.12	0.06 to 0.18	< 0.001	0.13	0.05 to 0.20	0.001	0.08	−0.01 to 0.17	0.07
BMI (per 5 kg/m2)	0.05	−0.03 to 0.12	0.21	−0.08	− 0.15 to − 0.01	0.03	−0.10	− 0.19 to − 0.01	0.03	−0.05	− 0.17 to 0.06	0.37
CDSS sum score	0.04	0.02 to 0.05	< 0.001	0.03	0.02 to 0.05	< 0.001	0.01	−0.02 to 0.03	0.48	0.04	0.02 to 0.06	< 0.001
AIP	0.36	0.11 to 0.62	0.005	–	–	–	–	–	–	–	–	–
Glucose (mmol/L)	0.09	−0.01 to 0.19	0.07	–	–	–	–	–	–	–	–	–
Problematic use of drugs	0.21	−0.04 to 0.45	0.097	–	–	–	–	–	–	–	–	–

*Note: suPAR* soluble urokoinase Plasminogen Activator Receptor; *CI* Confidence Interval, β = beta coefficient, *hsCRP* high sensitivity C-reactive protein, *BMI* Body Mass Index, *AIP* Atherogenic Index of plasma calculated as log10(Triglycerids/HDL-cholesterol), *CDSS* Calgary Depression Scale for Schizophrenia (0–27), Problematic use of drugs = above defined cut-off values for Drug Use Disorders Identification Test (DUDIT) (≥2 for females and ≥ 6 for males)

^a^Mulitvariable regression model including all variables listed. The amount of explained variance by the model (adjusted R^2^) was 0.35 for whole sample, 0.20 in males and 0.37 in females

### Sensitivity analyses

The sensitivity analyses supported the main finding of the study. We found similar results for the associations between clinical factors and suPAR levels when CDSS sum score was substituted by CDSS cutoff score of ≥6 and subsequently PANSS depressed factor (for further details see Supplementary Table A, Additional File 1). Studying the separate CDSS items (C1-C9), for females there were positive associations between suPAR and the CDSS items C3 (self depreciation), C4 (guilty ideas of reference), C5 (pathological guilt), C6 (morning depression), C7 (early wakening) and C8 (suicide). For C1 (self described depression) and C2 (hopelessness) the associations were borderline significant and there was no association between suPAR levels and C9 (observed depression), the only item based on the clinicians interpretation. For males neither of the items were significantly associated with suPAR levels (Table 4).

**Table 4:** Associations between CDSS and suPAR stratified by sex

	Males (*n* = 91)			Females (*n* = 72)		
Items of CDSS interview	β^a^	CI	*p*	β^a^	95% CI	*p*
C1: Self described depression	−0.02	− 0.13 to 0.09	0.75	0.20	0.05 to 0.35	0.012
C2: Hopelessness	0.04	−0.08 to 0.16	0.47	0.20	0.05 to 0.36	0.011
C3: Self depreciation	0.03	−0.08 to 0.13	0.61	0.20	0.07 to 0.32	0.002
C4: Guilty ideas of reference	0.19	0.03 to 0.35	0.02	0.21	0.06 to 0.36	0.006
C5: Pathological guilt	0.09	−0.09 to 0.26	0.34	0.22	0.10 to 0.35	0.001
C6: Morning depression	0.01	−0.12 to 0.15	0.86	0.22	0.07 to 0.36	0.004
C7: Early wakening	0.07	−0.05 to 0.19	0.27	0.18	0.06 to 0.31	0.005
C8: Suicide	−0.17	− 0.34 to − 0.00	0.045	0.31	0.13 to 0.48	0.001
C9: Observed depression	0.06	−0.10 to 0.22	0.44	0.13	−0.07 to 0.32	0.21

*Note: suPAR* soluble urokinase Plasminogen Activator Receptor (ng/ml), *CDSS* Calgary Depression Scale for Schizophrenia (0–27), *CI* Confidence Interval, β = beta coefficient, *CI* confidence interval

^a^Adjusted for age, current tobacco smoking, high sensitivity C-reactive protein and body mass index

Levels of hsCRP and depression measures (CDSS sum score, CDSS ≥6 and PANSS depressed factor) were not significantly associated (see Supplementary Text 2, Additional File 5). However, when stratifying by sex, there was a borderline significant association between hsCRP and CDSS sum score in females (beta 0.07, 95% CI 0.01 to 0.13) but not in males. When adjusting for BMI the association between hsCRP and CDSS sum score in females became clearly non-significant for females as well.

**Fig. 1:**
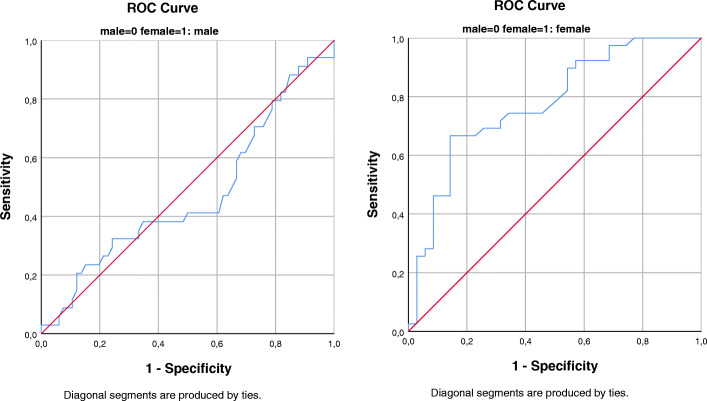
Receiver Operating Curves for suPAR vs depression. ROC curve suPAR vs depression defined as CDSS sum-score ≥ 6, stratified by sex. Males: Positive cases *n* = 34, negative cases *n* = 66. AUC = 0.47 (95% CI: 0.35 to 0.60). Females: Positive cases *n* = 39, negative cases *n* = 35. AUC = 0.78 (95% CI: 0.67 to 0.88)

We found that suPAR levels could predict depression (CDSS ≥6) in participants with schizophrenia to a limited extent only (Fig. 1).

## Discussion

In the current study we investigated the association between LGI by suPAR levels and clinical factors in schizophrenia. In multivariate analyses we found that sex, age, current tobacco smoking, BMI, hsCRP and depressive symptoms were significantly associated with levels of suPAR. However, we found an interaction between sex and CDSS, with positive association between CDSS sum score and suPAR levels only in females. In contrast, LGI reflected by hsCRP was not associated with depressive symptoms in schizophrenia.

To our knowledge, the current study is the first to investigate the relationship between the LGI marker suPAR and clinical symptoms in schizophrenia taking several potential confounders into account. Our results are not surprising as prior studies have shown associations between other pro-inflammatory cytokines and symptom severity in schizophrenia [44] and depressive symptoms in first episode psychosis [45]. Also, a recent study indicated that a decrease in Interleukin-6, a pro-inflammatory cytokine, was associated with a decrease in depressive symptoms in first episode schizophrenia patients [46]. Moreover, results from non-schizophrenia samples show an association between suPAR levels and depression [21–24].

In schizophrenia, prevalence of depression ranges from 30 to 60% [47–49] and with great variation between different subpopulations. Depression in our sample was comparable to these levels, also the female overrepresentation of depressive symptoms was in line with studies in the general population, [50, 51]. The sensitivity-analysis, substituting every single CDSS item with the sum score, demonstrated that no particular item or item cluster drove the association with suPAR levels in females.

Interestingly, suPAR levels were significantly associated with depressive symptoms in females only, while a follow-up study to Bot et al. (2015) patients with MDD indicated a positive association between suPAR levels and depression in males only [21, 52]. We are not able to explain this difference fully, but Ramsey et al. 2016 analyzed 171 different proteins in serum and included MDD per diagnosis, while in our study, we investigated primarily suPAR, and adjusted for hsCRP in plasma in participants with schizophrenia diagnose when measuring depressive symptoms (not MDD per diagnosis).

As increased suPAR levels reflect inflammation and are found to be associated with endothelial dysfunction, one could speculate that there is an association between suPAR levels and neuro-inflammation through endothelial cell dysfunction in the microvasculature of the brain [10, 53] as well as impaired neuro repair [13].

Depressive symptoms appear to play a part in the transition to first episode psychosis and seem to be a predictive factor of the outcome of schizophrenia [49]. Immune system aberrancies are associated with both schizophrenia and depression [8]. Our results indicate immune pathology is associated with depressive symptoms in females with schizophrenia. However, the results are explorative and need to be confirmed. The ROC analyses showed that suPAR could not predict depression in females with schizophrenia at a high enough level to use it as a sole biomarker, yet the strong association with depression should be further investigated in schizophrenia as suPAR could have potential as an early indicator of poorer outcome.

Sex, age, smoking and BMI are considered relevant adjustment factors in suPAR studies, and the current full sample multivariate analysis revealed that these variables were significantly associated with suPAR levels. The association between BMI and suPAR was negative in the multivariable analysis. Possible explanations for these findings are that BMI does not accurately reflect fat distribution, and we adjusted for hsCRP, which is a marker associated with central fat related inflammation as well as acute infection [54]. Also, the suPAR gene (PLAUR) is found to be upregulated in visceral fat of non-obese participants with mood disturbances and/or anxiety. However, it is uncertain to what degree circulating uPAR is affected by this, and the statistical power was low [17]. There were no statistically significant associations between suPAR and the proxies for CVD risk and Diabetes Mellitus Type II in the multivariate regression in our sample, plus we adjusted for hsCRP (a risk factor for CVD). This indicates that the association between suPAR and depression is strong, even in the presence of somatic disease.

The result of this study should be interpreted within its limitations; the sample size, especially when stratifying by sex, limited our possibilities to examine more factors with possible association with suPAR. It is also important to emphasize the exploratory nature of our study. Our focus was on the schizophrenia diagnosis, and we had only symptom measures for depression. However, the association between depressive symptoms and suPAR levels was highly significant in females and the sensitivity analyses revealed similar results. Including a naturalistic sample increased the risk of comorbid somatic diseases and medication affecting the immune system and thus may bias the results of our study. However, schizophrenia patients are a heterogeneous group with more prevalent comorbidity and medication compared to the healthy population [55]. The participants were recruited over a lengthy period and prevalence of some characteristics may have changed over this period, e.g. smoking habits, attention to healthy diet and physical activity.

When it comes to strengths, it is worth noting that our study consisted of a relatively large, well-characterized and heterogeneous sample. Thus, we were able to adjust for many of the important factors associated with suPAR. Our study included participants of both sexes and participants with known use of alcohol and drugs, thus being a naturalistic sample and reducing selection bias.

## Conclusion

We found that depressive symptoms in female patients with schizophrenia were significantly associated with suPAR levels after adjusting for confounding factors and inflammation by hsCRP. Our results suggest that immune processes measured by suPAR but not hsCRP, could be involved in the psychopathology in females with schizophrenia and depressive symptoms. Larger and longitudinal studies are warranted to confirm the present findings and identify the specific immune mechanisms related to elevated suPAR levels in schizophrenia.

## Supplementary Information

**Additional file 1: Table A** Sensitivity analysis: Associations between depressive symptoms and suPAR, multivariable regression analyses in participants with schizophrenia.

**Additional file 2: Table B1** Internal reliability of the Norwegian version of the Calgary Depression Scale for Schizophrenia. **Table B2** Internal reliability of the Norwegian version of the Calgary Depression Scale for Schizophrenia, by sex.

**Additional file 3:.** Metabolic risk associated with antipsychotic medication.

**Additional file 4: Appendix Text 1**: Associations between explored psychiatric symptoms.

**Additional file 5: Appendix Text 2** - Exploration of the relationship between hsCRP and depressive symptoms beyond CDSS sum score.

## Data Availability

The dataset generated and analyzed during the current study is not publicly available. This is due to the sensitive nature and as such the availability is restricted and regulated by Norwegian Laws and EC laws (GDPR). Upon reasonable request data availability will be considered according to current legislation on privacy and personal data protection regulations.

## Abbreviations

AIP: Atherogenic index of plasma
AUC: Area under the curve
AUDIT: Alcohol use disorder identification test
beta: Beta coefficient
BMI: Body mass index
CDSS: Calgary depression scale for schizophrenia
CI: Confidence intervals
CNS: Central nervous system
CRP: C-reactive protein
CVD: Cardiovascular disease
DDD: Defined daily doses
DSM: Diagnostic and statistical manual of mental disorders
DUDIT: Drug use disorder identification test
EPHAPS: Effects of physical activity in psychosis
HDL: High density lipoproteins
hsCRP: High sensitivity C-reactive protein
IQR: Interquartile range
kg: Kilogram
L: Liter
LGI: Low grade inflammation
m: Meter
MDD: Major depressive disorder
mg: Milligram
ml: Milliliter
mmol: Millimole
ng: Nanogram
NORMENT: Norwegian centre for mental disorder research
PANSS: Positive and negative syndrome scale
ROC: Receiver operating characteristic
SCID-I: Structured clinical interview for DSM-IV
SD: Standard deviation
suPAR: Soluble urokinase plasminogen activator receptor
TOP: Thematically organized psychosis
UCLA: University of California Los Angeles
uPAR: Urokinase plasminogen activator receptor
WHO: World health organization
β: Beta coefficient
