# Supplementary material for: Exploring low grade inflammation by soluble urokinase plasminogen activator receptor levels in schizophrenia: a sex-dependent association with depressive symptoms
Source: BMC Psychiatry. 2021 Oct 26;21:527. doi: 10.1186/s12888-021-03522-6 (PMC8547032; doi:10.1186/s12888-021-03522-6)
Supplement: Supplementary file 1 — Additional file 1: Table A Sensitivity analysis: Associations between depressive symptoms and suPAR, multivariable regression analyses in participants with schizophrenia. [file 12888_2021_3522_MOESM1_ESM.docx]

| **Table A**  **Sensitivity analysis: Associations between depressive symptoms and suPAR, multivariable regression analyses in participants with schizophrenia** | | | | | | |
| --- | --- | --- | --- | --- | --- | --- |
|  | Multivariable regression^a^ | | | Multivariable regression^b^ | | |
|  | β | 95% CI | p | β | 95% CI | p |
| Female sex | 0.29 | 0.14 to 0.44 | <0.001 | 0.24 | 0.08 to 0.39 | 0.003 |
| Age by 10 years | 0.08 | 0.02 to 0.14 | 0.010 | 0.10 | 0.04 to 0.16 | 0.002 |
| Tobacco smoking | 0.31 | 0.16 to 0.45 | <0.001 | 0.31 | 0.16 to 0.46 | <0.001 |
| hsCRP [mg/L] | 0.13 | 0.07 to 0.19 | <0.001 | 0.13 | 0.07 to 0.19 | <0.001 |
| BMI by 5 kg/m^2^ | -0.07 | -0.14 to 0.04 | 0.07 | -0.09 | -0.16 to -0.01 | 0.02 |
| CDSS≥6 | 0.25 | 0.10 to 0.40 | 0.001 | - | - | - |
| PANSS depressed factor | - | - | - | 0.04 | 0.01 to 0.06 | 0.003 |
| *Note:* suPAR= soluble urokoinase Plasminogen Activator Receptor; β = beta coefficient, CI=Confidence Interval, hsCRP=high sensitivity C-reactive protein, BMI=Body Mass Index, CDSS=Calgary Depression Scale for Schizophrenia.  ^a^Mulitvariate regression model, replacing CDSS sum score by CDSS ≥ 6. N=163. Adjusted R^2^=0.32. ^b^Mulitvariable regression model, replacing CDSS sum score by PANSS depressed factor. N=168, Adjusted R^2^=0.31 | | | | | | |
