# Supplementary material for: Exploring low grade inflammation by soluble urokinase plasminogen activator receptor levels in schizophrenia: a sex-dependent association with depressive symptoms
Source: BMC Psychiatry. 2021 Oct 26;21:527. doi: 10.1186/s12888-021-03522-6 (PMC8547032; doi:10.1186/s12888-021-03522-6)
Supplement: Supplementary file 2 — Additional file 2: Table B1 Internal reliability of the Norwegian version of the Calgary Depression Scale for Schizophrenia. Table B2 Internal reliability of the Norwegian version of the Calgary Depression Scale for Schizophrenia, by sex. [file 12888_2021_3522_MOESM2_ESM.docx]

| **Table B1**  **Internal reliability of the Norwegian version of the Calgary Depression Scale for Schizophrenia** | | | | |
| --- | --- | --- | --- | --- |
| Item | Mean value  if item deleted | | Item total-correlation | Cronbach’s alpha coefficient with item deleted |
| C1 Self described depression | | 4.48 | 0.74 | 0.85 |
| C2: Hopelessness | | 4.60 | 0.75 | 0.85 |
| C3: Self-depreciation | | 4.50 | 0.71 | 0.85 |
| C4: Guilty ideas of reference | | 5.11 | 0.52 | 0.87 |
| C5: Pathological guilt | | 4.96 | 0.66 | 0.86 |
| C6: Morning depression | | 4.98 | 0.59 | 0.86 |
| C7: Early wakening | | 4.86 | 0.48 | 0.87 |
| C8: Suicide | | 5.02 | 0.59 | 0.86 |
| C9: Obeserved depression | | 4.98 | 0.52 | 0.87 |
| *Note:* Data missing CDSS: n=13 in whole sample  Chronbach’s alpha coefficient whole sample = 0.87 (standardized = 0.88) (n = 174) | | | | |

|  |  |  |  |  |  |  |
| --- | --- | --- | --- | --- | --- | --- |
| **Table B2**  **Internal reliability of the Norwegian version of the Calgary Depression Scale for Schizophrenia, by sex** | | | | | | |
| Item | Mean value  if item deleted | Item total-correlation | Cronbach’s alpha coefficient  if item deleted | Mean value  if item deleted | Item total-correlation | Cronbach’s alpha coefficient  if item deleted |
| C1 Self described depression | 3.41 | 0.76 | 0.72 | 5,93 | 0.77 | 0.89 |
| C2: Hopelessness | 3.56 | 0.75 | 0.73 | 6,01 | 0.77 | 0.89 |
| C3: Self-depreciation | 3.55 | 0.58 | 0.75 | 5,78 | 0.79 | 0.89 |
| C4: Guilty ideas of reference | 4.12 | 0.14 | 0.80 | 6,45 | 0.69 | 0.90 |
| C5: Pathological guilt | 4.03 | 0.42 | 0.78 | 6,22 | 0.74 | 0.89 |
| C6: Morning depression | 3.90 | 0.46 | 0.77 | 6,45 | 0.68 | 0.90 |
| C7: Early wakening | 3.82 | 0.32 | 0.80 | 6,26 | 0.58 | 0.91 |
| C8: Suicide | 4.00 | 0.30 | 0.79 | 6,39 | 0.73 | 0.90 |
| C9: Obeserved depression | 3.93 | 0.58 | 0.76 | 6,41 | 0.45 | 0.91 |
| *Note:* Data missing CDSS: males missing n=8, females missing n=5 missing  Chronbach’s alpha coefficient for males = 0.79 (standardized = 0.78) (n = 100)  Chronbach’s alpha coefficient for females = 0.91 (standardized = 0.91) (n = 74) | | | | | | |

|  |
| --- |
