# Supplementary material for: Exploring low grade inflammation by soluble urokinase plasminogen activator receptor levels in schizophrenia: a sex-dependent association with depressive symptoms
Source: BMC Psychiatry. 2021 Oct 26;21:527. doi: 10.1186/s12888-021-03522-6 (PMC8547032; doi:10.1186/s12888-021-03522-6)
Supplement: Supplementary file 3 — Additional file 3:. Metabolic risk associated with antipsychotic medication. [file 12888_2021_3522_MOESM3_ESM.docx]

| **Table C**  **Metabolic risk associated with antipsychotic medication** | | | |
| --- | --- | --- | --- |
| Antipsychotic medication | Low  metabolic risk | Moderate metabolic risk | High  metabolic risk |
| Aripiprazole | x |  |  |
| Clozapine |  |  | x |
| Paliperidone |  | x |  |
|  |  |  |  |
| Risperidone |  | x |  |
| Olanzapine |  |  | x |
| Chlorprothixene |  | x |  |
| Quetiapine |  | x |  |
| Amisulpiride | x |  |  |
| Perphenazine | x |  |  |
| Flupentixol |  | x |  |
| Haloperidol | x |  |  |
| Zuclopenthixole |  | x |  |
| Ziprasidone | x |  |  |
| Sertindole |  | x |  |
| *Note:* Data derived from Recommendations of the Polish Psychiatric Association (Wichiniak et al 2018) | | | |
