# Supplementary material for: Exploring low grade inflammation by soluble urokinase plasminogen activator receptor levels in schizophrenia: a sex-dependent association with depressive symptoms
Source: BMC Psychiatry. 2021 Oct 26;21:527. doi: 10.1186/s12888-021-03522-6 (PMC8547032; doi:10.1186/s12888-021-03522-6)
Supplement: Supplementary file 4 — Additional file 4: Appendix Text 1: Associations between explored psychiatric symptoms. [file 12888_2021_3522_MOESM4_ESM.docx]

**Appendix Text 1: Associations between explored psychiatric symptoms**

To make sure the symptom domains did not overlap excessively, we explored the relationship between them. In the whole sample CDSS sum score was significantly related to other measures of depression (CDSS ≥ 6 (beta 8.64, 95% CI. 7.74 to 9.54) and PANSS depressed factor (beta 1.18, 95% CI 1.02 to 1.34)) as well as positive symptoms (PANSS positive) (beta 0.26, 95% CI 0.11 to 0.41). There was no significant association between CDSS sum score and negative symptoms. When stratifying by sex the CDSS sum score was significantly associated with other measures of depression (CDSS ≥ 6 (beta 7.14, 95% CI 6.31 to 7.96) and PANSS depressed factor (beta 0.91, 95% CI 0.72 to 1.11)), but there was no significant association with positive or negative symptoms for males. For females CDSS sum score was significantly associated with both the depression measures (CDSS ≥ 6 (beta 10.00, 95% CI 8.28 to 11.73) and PANSS depressed factor (beta 1.42, 95% CI 1.13to 1.71)) as well as positive symptoms (beta 0.38, 95% CI 0.14 to 0.63), but there was no association with negative symptoms.

The associations between CDSS sum score, CDSS cutoff ≥ 6 and PANSS depressed factor were strong and significant in the entire patient sample and for sexes separately. A low to modest overlap between CDSS sum score and positive and negative PANSS scores have previously been reported [1]. In our study, the depressive symptoms correlated with positive symptoms in the whole sample and in females only when stratified by sex. There was no correlation with negative symptoms, indicating that we measured depressive symptoms and not negative symptomatology. The Cronbach’s alpha for the CDSS showed high internal consistency of this instrument.

1. Muller MJ, Brening H, Gensch C, Klinga J, Kienzle B, Muller KM: **The Calgary Depression Rating Scale for schizophrenia in a healthy control group: psychometric properties and reference values**. *J Affect Disord* 2005, **88**(1):69-74, doi:10.1016/j.jad.2005.04.005.
