# Supplementary material for: Exploring low grade inflammation by soluble urokinase plasminogen activator receptor levels in schizophrenia: a sex-dependent association with depressive symptoms
Source: BMC Psychiatry. 2021 Oct 26;21:527. doi: 10.1186/s12888-021-03522-6 (PMC8547032; doi:10.1186/s12888-021-03522-6)
Supplement: Supplementary file 5 — Additional file 5: Appendix Text 2 - Exploration of the relationship between hsCRP and depressive symptoms beyond CDSS sum score. [file 12888_2021_3522_MOESM5_ESM.docx]

**Appendix Text 2 - Exploration of the relationship between hsCRP and depressive symptoms beyond CDSS sum score**

We found no significant association of hsCRP between neither CDSS ≥ 6 (beta 0.08, 95% CI -0.37 to 0.53) nor PANSS depressed factor (beta 0.02, 95% CI -0.04 to 0.09) for the whole sample. Again, when stratifying we found no significant associations of hsCRP between neither CDSS ≥ 6 (beta -0.49, 95% CI -1.08 to 0.11) nor PANSS depressed factor (beta -0.04, 95% CI -0.13 to 0.05) for males and neither CDSS ≥ 6 (beta 0.70, 95% CI -0.01 to 1.41) nor PANSS depressed factor (beta 0.09, 95% CI -0.01 to 0.20) for females.
